# Potential mechanism underlying HXSJ decoction in the treatment of venous leg ulcers: based on the association between venous leg ulcers and ferroptosis

**DOI:** 10.1186/s12906-025-05184-3

**Published:** 2025-12-30

**Authors:** Sunfeng Pan, Lie Xiong, Jiakun Li, Zhenjun Wang, Yujuan Su, Gaofeng Fang, Minda Zhu, Hanqiang Shi, Jiayan Li, Zhaoyan Liu, Chunmao Han, Yanbo Shi

**Affiliations:** 1 Zhejiang University School of Medicine, Hangzhou, 310006 China; 2 Department of Burn and Plastic Surgery, Zhejiang Chinese Medical University Affiliated Jiaxing Traditional Chinese Medicine Hospital, Jiaxing, 314000 China; 3 Central Laboratory of Molecular Medicine Research Center, Zhejiang Chinese Medical University Affiliated Jiaxing Traditional Chinese Medicine Hospital, Jiaxing, 314000 China; 4 Jiaxing Burn and Wound Repair Therapy Center, Jiaxing, 314000 China; 5 Jiaxing Key Laboratory of Diabetic Angiopathy Research, Jiaxing, 314000 China; 6 Zhejiang Chinese Medical University, Hangzhou, 310053 China; 7 Department of Burns and Wound Repair, the Second Affiliated Hospital of Zhejiang University School of Medicine, Hangzhou, 310009 China

**Keywords:** Venous leg ulcer, Ferroptosis, Chinese herbal compound, Iron overload, HUVECs

## Abstract

**Background:**

Venous leg ulcer (VLU) is among the most severe clinical manifestations of chronic venous disease (CVD) and imposes substantial burdens on both patients and society. VLU pathogenesis is closely associated with the impairment of vascular endothelial cells. In this study, tissue samples from the patients’ s skins around wound at different stages of CVD were collected during operation and used to elucidate the involvement of ferroptosis in the pathogenesis of VLU. Also the potential mechanism through which Huoxue Shengji (HXSJ) decoction alleviates ferroptosis in human umbilical vein endothelial cells (HUVECs) was investigated.

**Methods:**

During surgical procedure such as great saphenous vein high ligation and stripping surgery, matched skin tissues from the normal, hyperpigmentation (HPT), lipodermatosclerosis (LDS), and VLU regions were collected from 10 patients with VLU, and the levels of iron and glutathione peroxidase 4 (GPX4) were quantified to evaluate ferroptosis. In vitro, HUVECs were used to iron overload induction using exogenous 100 µM ferric ammonium citrate (FAC) or 100 µM hemin, or ferroptosis induction with 10 µM erastin, and treated with 10 µg/ml HXSJ decoction for 24 h. Subsequently, lipid peroxidation (LPO) damage, mitochondrial function, and key genes involved in ferroptosis were assessed.

**Results:**

Iron deposition in the affected skin of patients with CVD gradually increased before progression to VLU and significantly decreased during the VLU stage. Moreover, GPX4 expression increased significantly in the HPT stage but was gradually suppressed with further deterioration of CVD. The in vitro results indicated that in both the iron overload and ferroptosis models, HXSJ decoction effectively upregulated the expression of nuclear factor erythroid 2-related factor 2 (Nrf2), solute carrier family 7 member 11 (SLC7A11, xCT), and GPX4, which was accompanied by the inhibition of malondialdehyde and protein carbonylation production, the alleviation of ferrous ion accumulation, and the restoration of mitochondrial function.

**Conclusions:**

This study demonstrated that iron accumulation-mediated inactivation of GPX4 serves as a crucial mechanism in VLU formation through ferroptosis induction. Notably, the therapeutic mechanism through which HXSJ decoction alleviates ferroptosis involves Nrf2/xCT/GPX4 pathway activation and a reduction in ferrous ion accumulation. These findings may provide novel insights into VLU pathogenesis and give possible reason for developing traditional Chinese medicine therapies targeting ferroptosis and VLUs.

**Graphical Abstract:**

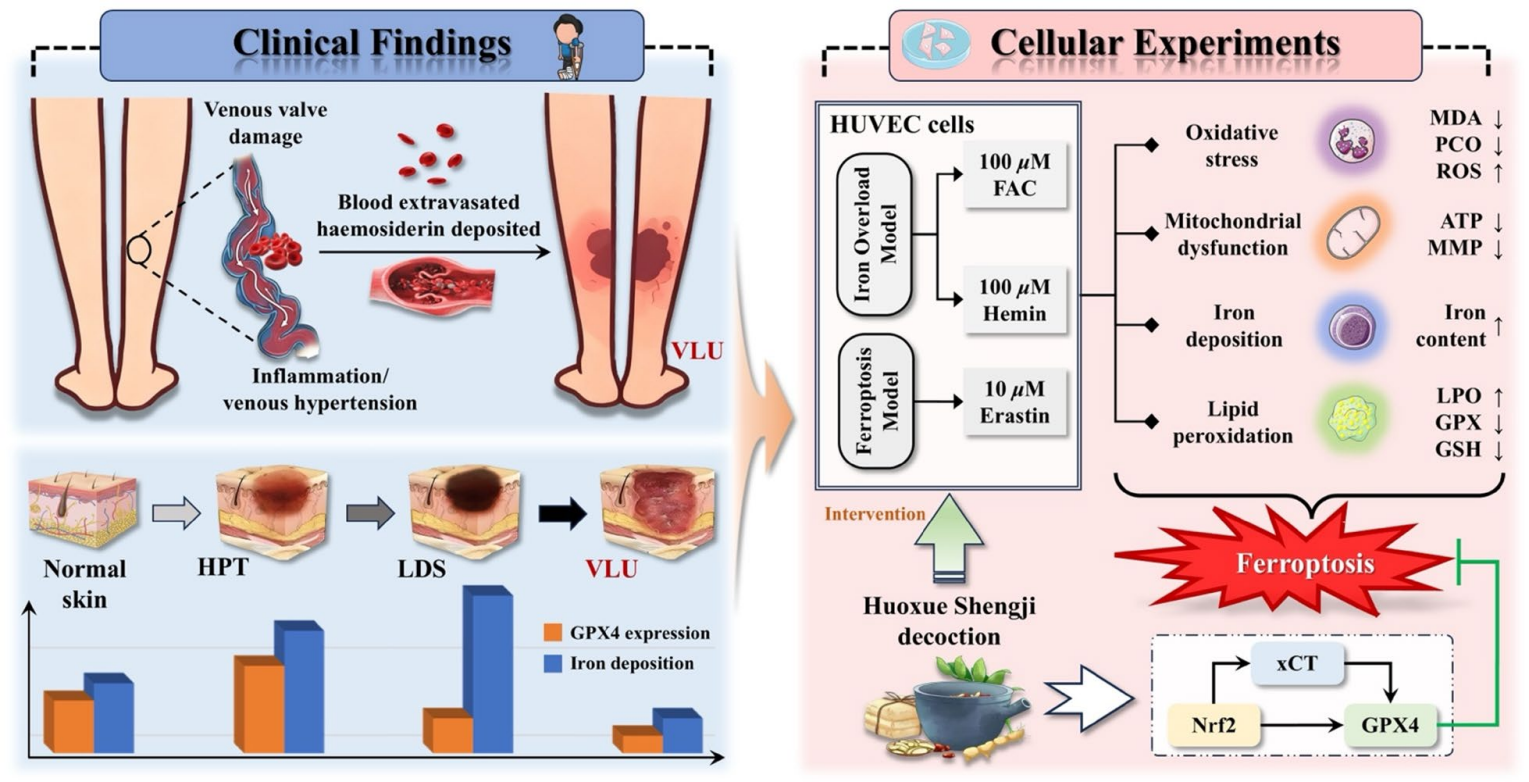

**Supplementary Information:**

The online version contains supplementary material available at 10.1186/s12906-025-05184-3.

## Background

Venous leg ulcer (VLU) is a chronic, recurrent skin defect of the lower extremity caused by chronic venous insufficiency (CVI), primarily resulting from sustained venous hypertension due to venous reflux, obstruction, or combined pathology. This condition leads to capillary damage, impaired local microcirculation, and progressive tissue hypoxia, ultimately triggering ulcer formation [1]. It is among the most severe clinical manifestations of CVD and accounts for approximately 70% of all lower limb ulcers [1]. VLUs not only cause chronic pain and disability in patients but also impose significant financial burdens and labour force depletion on society [2]. These challenges are further compounded by impaired wound healing capacity and high recurrence rates [3]. Thus, conducting in-depth research on VLU holds immense significance.

Clinically, VLU progression can be primarily categorized into three stages: hyperpigmentation (HPT), lipodermatosclerosis (LDS), and ultimately, VLU. In normal physiology, wound healing is an intricately orchestrated biological process that can be broadly categorized into four stages: haemostasis, inflammation, proliferation, and remodelling [4]. Angiogenesis plays a pivotal role in the wound healing process by facilitating the formation of granulation tissue [5]. Conversely, impairment of vascular endothelial cells (VECs) can lead to ulcer development and delayed wound closure, which is considered a contributing factor to VLU [6].

Recent studies have shown that hyperpigmentation (HPT) and lipodermatosclerosis (LDS) represent the initial stages of venous leg ulcer (VLU) and are characterized by specific pathological changes, including iron deposition [7–9]. Iron deposition is a key factor in the impaired healing of chronic skin ulcers (CSUs), including VLUs [10, 11]. The relationship between iron deposition and VLU pathogenesis can be explained in two ways. On the one hand, iron deposition blocks the exchange of substances between capillaries and tissues, leading to cellular hypoxia and metabolic disruption [12]. On the other hand, iron deposition mediates the release of inflammatory factors [13, 14]. However, we observed that some CVD patients tend to stabilize at the HPT stage and do not progress to LDS. Once the disease progresses to the LDS stage, VLUs will develop rapidly. Recent research has established a direct correlation between VEC ferroptosis and CSUs [15, 16]. Excessive iron, particularly ferrous iron, induces lipid peroxidation (LPO) damage via the Fenton reaction, thereby inducing ferroptosis, a form of programmed cell death dependent on iron and LPO [17]. These findings suggest a potential correlation between ferroptosis and VLUs, which warrants further investigation.

Negative pressure wound therapy (NPWT) is a commonly used clinical treatment method that promotes VLU healing. Nevertheless, NPWT has some problems, such as poor drainage and a risk of infection [18]. For this reason, NPWT with instillation (NPWTi) has gained increasing attention in recent years [19]. Our previous clinical study confirmed that NPWTi with Huoxue Shengji (HXSJ) decoction effectively promotes angiogenesis and wound healing [20]. HXSJ decoction is a traditional Chinese medicinal compound used externally for treating VLUs and has been clinically applied by our research group for more than a decade.

Here, we investigated the involvement of ferroptosis in skin tissue at different stages of VLU progression and explored the potential mechanisms of HXSJ decoction using iron overload and ferroptosis models in human umbilical vein endothelial cells (HUVECs).

## Methods

### Experimental environment and procedures

#### Tissue sample collection

**Fig. 1:**
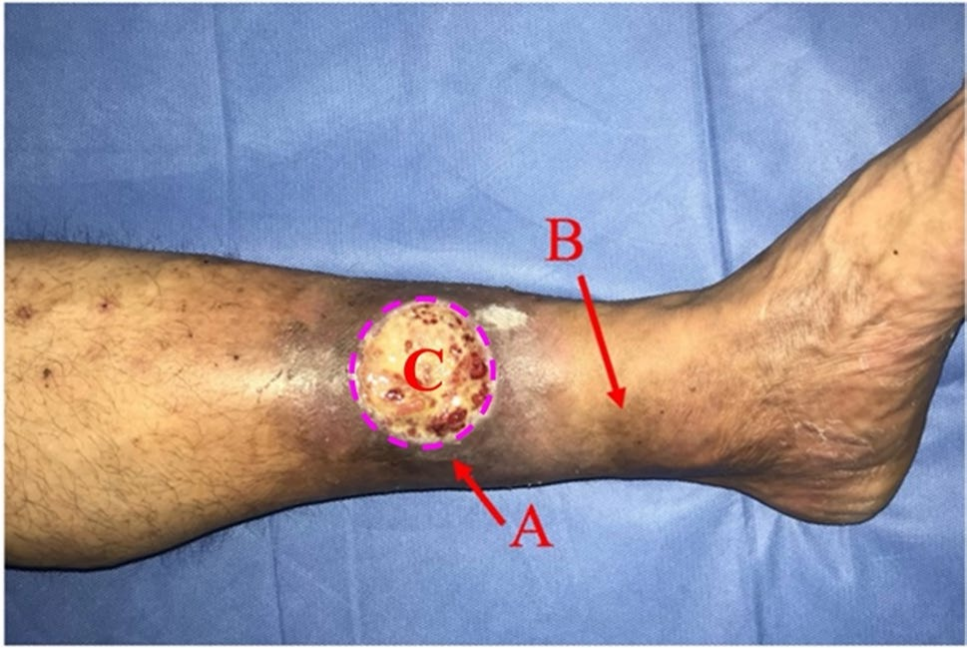
Schematic diagram of the VLU. **A**: LDS regions around the ulcer; **B**: HPT regions close to the ankle; **C**: Ulcer area

This study was conducted in accordance with the Declaration of Helsinki and approved by the Ethics Committee of Jiaxing Traditional Chinese Medicine Hospital (REC reference number: 2019KY0454). Informed consent forms were signed by each patient included in the study. Ten VLU patients who were classified as C5 according to the CEAP classification system [21] and who were indicated for great saphenous vein high ligation and stripping surgery were included in this study. During surgical procedures, patients’s matched skin tissues around wound from the normal, HPT, LDS, and VLU regions were collected. VLU tissue was obtained during the process of ulcer debridement by sampling from the junction between the ulcer and normal skin, including the base of the ulcer with a thickness of 3 mm. During the great saphenous vein high ligation and stripping surgery, 3 mm × 5 mm full-thickness skin was obtained from the HPT and LDS regions, respectively. The incisions were subsequently used for the stripping of the varicose veins. A full-thickness normal skin sample (3 mm×20 mm) was obtained from the inguinal incision, which was subsequently used for high ligation of the great saphenous vein. The typical HPT and LDS regions are illustrated in Fig. 1. All the samples were rinsed with saline, weighed, and embedded or frozen rapidly in liquid nitrogen for storage at −80 °C.

#### Cell culture and grouping

HUVECs were obtained from the National Infrastructure of Cell Line Resources (Beijing, China) and cultured in DMEM (10569010; Gibco, USA) supplemented with 10% foetal bovine serum (FBS; 10100147; Gibco, USA) and 100 U/ml penicillin sodium- 100 µg/ml streptomycin sulfate (B540732; Sangon, China). The cells were maintained in a humidified incubator (370, Thermo, USA) at 37 °C with 5% CO_2_.

Iron overload models were established using 100 µM ferric ammonium citrate (FAC, A500061, Sangon, China) or 100 µM hemin (H140872, Aladdin, China) to simulate simple iron overload or haemoglobin exudation, respectively. Erastin (10 µM; HY-15763, MCE, USA) was used to establish a ferroptosis model. The experiment was conducted following a 24 h intervention with HXSJ decoction and modelling drugs simultaneously.

#### Preparation of Huoxue Shengji Decoction

The components of the HXSJ decoction are presented in Table 1. *Astragali Radix* (15 g, Cat. 170320), *Salviae Miltiorrhizae Radix et Rhizoma* (20 g, Cat. 170307), *Carthami Flos* (10 g, Cat. 170328) and *Glycyrrhizae Radix et Rhizoma* (6 g, Cat. 170301) were obtained from Anhui Jiayou Chinese Medicine Herb Pieces Co., Ltd. *Angelicae Sinensis Radix* (20 g, Cat. 170202) and *Paridis Rhizoma* (10 g, Cat. 170305) were obtained from Zhejiang Chinese Medical University Medical Pieces Co., Ltd. *Olibanum* (10 g, Cat. 170224), *Bletillae Rhizoma* (10 g, Cat. 170112), and *Myrrha* (10 g, Cat. 170230) were obtained from Jiaxing Oriental Chinese Medicine Decoction Pieces Co., Ltd. *Angelicae Dahuricae Radix* (10 g, Cat. 44170201) was obtained from Hangzhou Mintai Traditional Chinese Medicine Decoction Pipe Co., Ltd. The aforementioned Chinese herbal medicine was extracted by hot reflux 1 h with 1 L 80% ethanol 3 times, and the extract was concentrated through rotary evaporation. The paste was subsequently dissolved in 80% ethanol to achieve a concentration of HXSJ Decoction at 500 mg/ml, filtered through a 0.22 μm filter and stored at −20 °C for later use.

**Table 1:** Components of HXSJ Decoction

Name^1^	Latin binomial nomenclature	medicinal parts
Astragali Radix	*Astragalus membranaceus (Fisch.) Bge.*	radix
Salviae Miltiorrhizae Radix et Rhizoma	*Salvia miltiorrhiza Bge.*	radix and rhizoma
Carthami Flos	*Carthamus tinctorius L.*	flower
Glycyrrhizae Radix et Rhizoma	*Glycyrrhiza glabra L.*	radix and rhizoma
Angelicae Sinensis Radix	*Angelica sinensis (Oliv.) Diels*	radix
Paridis Rhizoma	*Paris polyphylla Smith var. yunnanensis (Franch.) Hand.-Mazz.*	rhizoma
Olibanum	*Boswellia carterii Birdw.*	resin
Bletillae Rhizoma	*Bletilla striata (Thunb.) Reichb.f.*	rhizoma
Myrrha	*Commiphora myrrha Engl.*	resin
Angelicae Dahuricae Radix	*Angelica dahurica (Fisch.ex Hoffm.) Benth.et Hook.f.*	radix

From the Chinese Pharmacopoeia (2020 edition)

### Evaluation methods

#### Prussian blue staining

The embedded samples were stained according to the instructions of the Prussian Blue Staining Kit (E670109, Sangon, China) and photographed under an upright microscope (Axio Scope. A1, Zeiss, Germany).

#### Immunohistochemistry (IHC)

Following the sequential steps of slicing, dewaxing, and antigen retrieval, the embedded samples were incubated with a GPX4 primary antibody (ab125066; Abcam, UK) and a goat anti-rabbit secondary antibody (PV-8000D; ZSGB-BIO, China). DAB staining was subsequently performed, and the samples were photographed under an upright microscope.

#### Cell viability assay

HUVECs were seeded into 96-well plates at 6 × 10^3^ cells/well. Briefly, after the intervention, 100 µl of medium supplemented with 10% CCK-8 reagent (E606335; Sangon, China) was added to each well, and the cells were subsequently incubated for 1 h. The absorbance at 450 nm was measured by a microplate reader. Cell viability = (*A*_experiment_-*A*_blank_)/(*A*_*control*_-*A*_blank_)×100%.

#### Biochemical analysis

The tissue samples were homogenized with saline, and the supernatant was collected after centrifugation. In accordance with the instructions of the Tissue Iron Content Assay Kit (BC4355, Solarbio, China), GPX Activity Assay Kit (D799618, Sangon, China), and BCA Protein Assay Kit (C503051, Sangon, China), the results were measured using a microplate reader (Multiskan GO, Thermo, USA), and quantified according to the protein concentration.

HUVECs were seeded into 6-well plates at 2 × 10^5^ cells/well. After intervention, cellular samples were harvested and analysed using a malondialdehyde (MDA) Content Assay Kit (D799762, Sangon, China), a protein carbonylation (PCO) Content Assay Kit (D799768, Sangon, China), a glutathione peroxidase (GPX) Activity Assay Kit, a glutathione (GSH) Content Assay Kit (D799614, Sangon, China), a Ferrous Iron Assay Kit (E-BC-K881-M, Elabscience, China), an ATP Assay Kit (S0026, Beyotime, China) and a BCA Protein Assay Kit. The results were measured using a microplate reader or a luminometer (GloMax 20/20; Promega, USA) and quantified according to the protein concentration.

#### Fluorescence staining assay

##### Intracellular reactive oxygen species (ROS) assay

The fluorescent probe 2’,7’-dichlorofluorescein diacetate (DCFH-DA, 35845, Sigma, USA) was employed to quantify intracellular ROS levels. After cellular entry, the deacetylated derivative of DCFH-DA is oxidized to a fluorescent product exhibiting green emission. In strict accordance with the instructions, HUVECs were incubated with 10 µM DCFH-DA at 37 °C for 30 min. After the cells were washed with PBS, fluorescence images were obtained using an inverted fluorescence microscope (Axio Observer D1, ZEISS, Germany) in the FITC channel.

##### **Lipid peroxidation (LPO) assay**

LPO was investigated using BODIPY™ 581/591 C11 (D3861, Thermo, USA), which converts red fluorescence to green fluorescence upon oxidation. Following the protocol described by Martinez et al. [22], HUVECs were incubated with 2.5 µM BODIPY™ 581/591 C11 at 37 °C for 30 min. After the cells were washed with PBS, fluorescence images were obtained using a fluorescence inverted microscope in the FITC and rhodamine channels.

##### **Mitochondrial membrane potential (MMP) assay**

MMP was investigated using JC-1 (C2006; Beyotime, China), which converts red fluorescence to green fluorescence as the MMP decreases. In brief, HUVECs were incubated with 10 µg/ml JC-1 at 37 °C for 30 min. After the cells were washed with buffer, fluorescence images were obtained using a fluorescence inverted microscope in the FITC and rhodamine channels.

#### Gene expression analysis

##### **Quantitative PCR (qPCR)**

HUVECs were seeded into 6-well plates and then harvested after intervention. Total RNA was extracted by RNAiso Plus (9109; Takara, Japan) and quantified using a spectrophotometer (Quickdrop, Molecular Devices, USA). cDNA was subsequently synthesized with a reverse transcription kit (RR036; Takara, Japan). qPCR was performed with a qPCR kit (RR820, Takara, Japan) on a real-time PCR system (7500, ABI, USA) at 95 °C for 30 s, 1 cycle; 95 °C for 5 s and 60 °C for 34 s, 40 cycles. β-Actin served as a reference, and the relative expression levels were calculated using the 2^−△△Ct^ method. The primer sequences are listed in Table 2.

**Table 2:** qPCR primer sequences

Gene	Forward sequence (5’- 3’)	Reverse sequence (5’- 3’)
*Nrf2*	AGTGTGGCATCACCAGAACA	TGTTTGACACTTCCAGGGGC
*SLC7A11*	CATCTCTCCTAAGGGCGTGC	TAGTGACAGGACCCCACACA
*GPX4*	CAGTGAGGCAAGACCGAAGT	CCGAACTGGTTACACGGGAA
*β-Actin*	CCTGGCACCCAGCACAAT	GGGCCGGACTCGTCATAC

*Nrf2* nuclear factor erythroid 2-related factor 2, *SLC7A11* solute carrier family 7 member 11, *GPX4* glutathione peroxidase 4, *β-*actin beta-actin

##### **Western blotting**

HUVECs were seeded into 6-well plates and then harvested after intervention. Total protein was extracted using RIPA lysis buffer (C500005; Sangon, China) supplemented with protease inhibitor (C600380; Sangon, China) and quantified with a BCA protein assay kit. Total protein (20 µg) was subsequently loaded onto a 10% SDS‒PAGE gel and transferred to a PVDF membrane (F619534; Sangon, China). After blocking, the membrane was incubated with anti-Nrf2 (ab92946; abcam, UK), anti-xCT (ab175186; abcam, UK), anti-GPX4 (ab125066; abcam, UK), and anti-GAPDH (ab181602; abcam, UK) primary antibodies at 4 °C overnight followed by a goat anti-rabbit (ab6721; abcam, UK) secondary antibody at RT for 1 h. Finally, enhanced chemiluminescence (ECL) reagent (C500044; Sangon, China) was used for visualization on a chemiluminescence imaging system (5200 Multi; Tanon, China).

### Statistical analysis

Statistical analysis was performed with SPSS software (version 23.0, USA). The data from clinical samples are presented as medians (lower quartile, upper quartile) and were analysed using multiple related sample nonparametric tests (Friedman test). The data from cell experiments are presented as the mean ± SD and were analysed using one-way ANOVA. *P* < 0.05 was considered to be statistical significance.

## Results

### Progression of chronic venous disease with iron accumulation and glutathione peroxidase suppression

This study included 10 patients with VLUs, and the basic information of each patient is shown in Table 3. Normal skin, HPT, LDS, and VLU samples were successfully collected from each patient, with no adverse events occurring during surgery. All patients achieved favourable therapeutic outcomes following standardized treatment.

**Table 3:** Basic information of each patient

No.	Gender	Age	Hb1Ac (%)	TG (mmol/L)	CHO (mmol/L)	ALT (U/L)	AST (U/L)	CREA (µmol/L)
1	Female	65	5.3	0.53	4.41	21	16	76
2	Female	68	6.7	0.99	2.56	17	17	59
3	Female	76	5.5	5.60	5.68	9	18	62
4	Male	58	5.7	1.00	3.89	14	18	68
5	Female	59	5.9	0.39	6.41	12	21	72
6	Female	65	5.2	1.43	4.44	10	17	71
7	Male	57	5.3	0.93	4.74	26	21	75
8	Male	66	6.5	2.37	3.07	23	15	87
9	Female	57	5.8	1.42	5.8	12	23	61
10	Male	46	5.0	1.83	3.53	29	26	63

*Hb1Ac* glycated haemoglobin A1c, *TG* triglyceride, *CHO* cholesterol, *ALT* alanine transaminase, *AST* aspartate transaminase, *CREA* creatinine

**Fig. 2:**
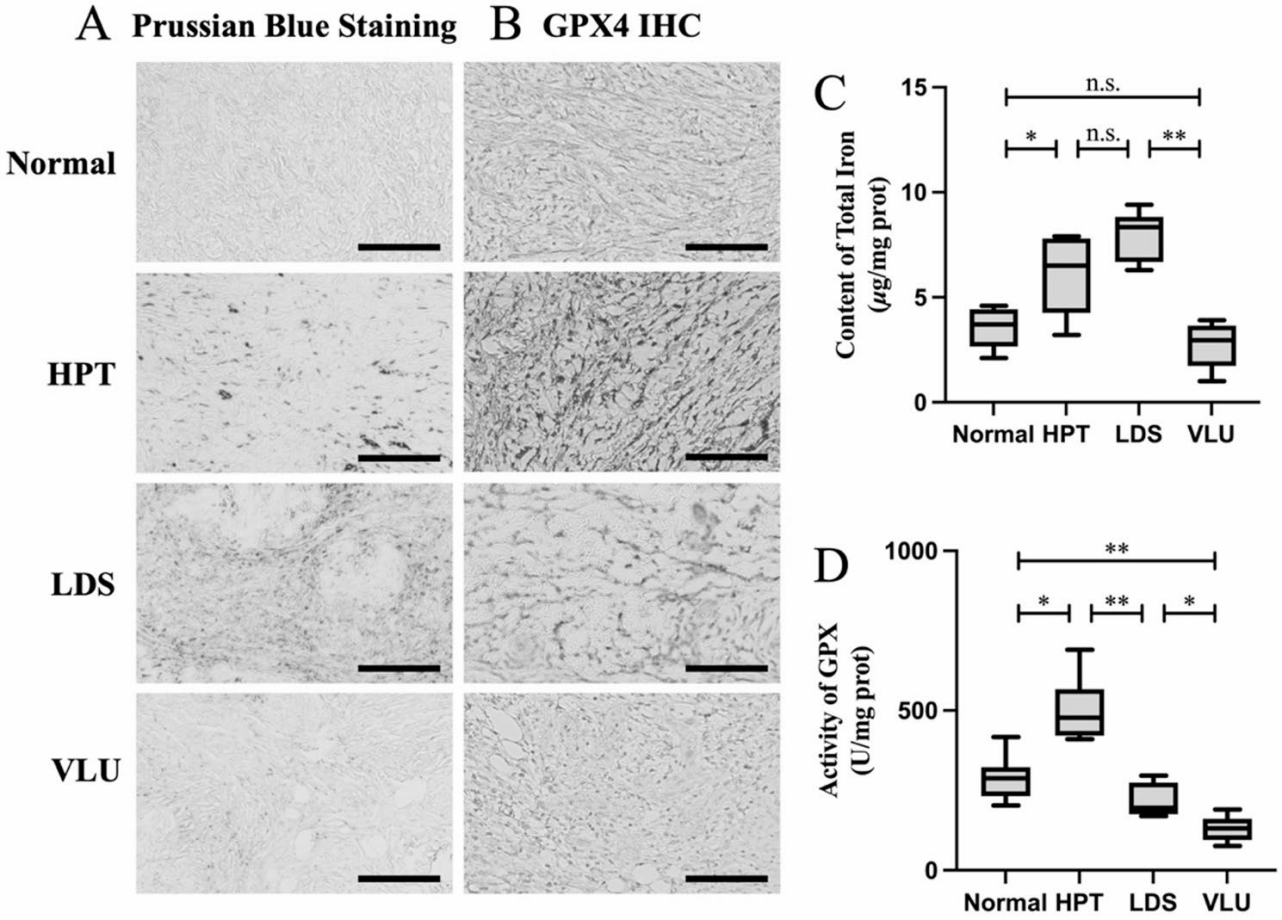
Iron accumulation and GPX suppression in distinct pathological regions of VLU patients. Prussian blue staining (**A**) and GPX4 IHC staining (**B**) of distinct pathological regions from a typical VLU patient; scale bar: 200 μm. Total iron (**C**) and GPX activity (**D**) in distinct pathological regions of VLU patients. **: *P* < 0.01; *: *P* < 0.05; n.s.: not significant.

Prussian blue staining, depicted in Fig. 2A for a typical patient, revealed that iron deposition in affected skin gradually increased as CVD progressed. However, there was an abrupt decrease in iron accumulation upon reaching the VLU stage. Consistent with these staining results, the results of the quantitative analysis of total iron content, shown in Fig. 2C, revealed significantly elevated levels in the HPT and LDS stages compared with those in the normal region (*P* < 0.05). However, a notable reduction was evident at the VLU stage compared with the LDS stage (*P* < 0.01). Furthermore, the IHC results for GPX4 from a typical patient are shown in Fig. 2B. GPX4 expression increased as CVD progressed to the HPT stage. However, with further deterioration to the LDS stage, GPX4 expression gradually decreased. In accordance with these IHC results, the quantitative analysis of GPX activity, illustrated in Fig. 2D, revealed a significant increase in the HPT region compared with that in normal skin (*P* < 0.05). As CVD progressed to the LDS stage, GPX activity decreased significantly compared with that in the HPT region (*P* < 0.01), and a further decline was observed when it reached the VLU stage (*P* < 0.05).

### Huoxue Shengji Decoction alleviates iron overload-induced damage to HUVECs

**Fig. 3:**
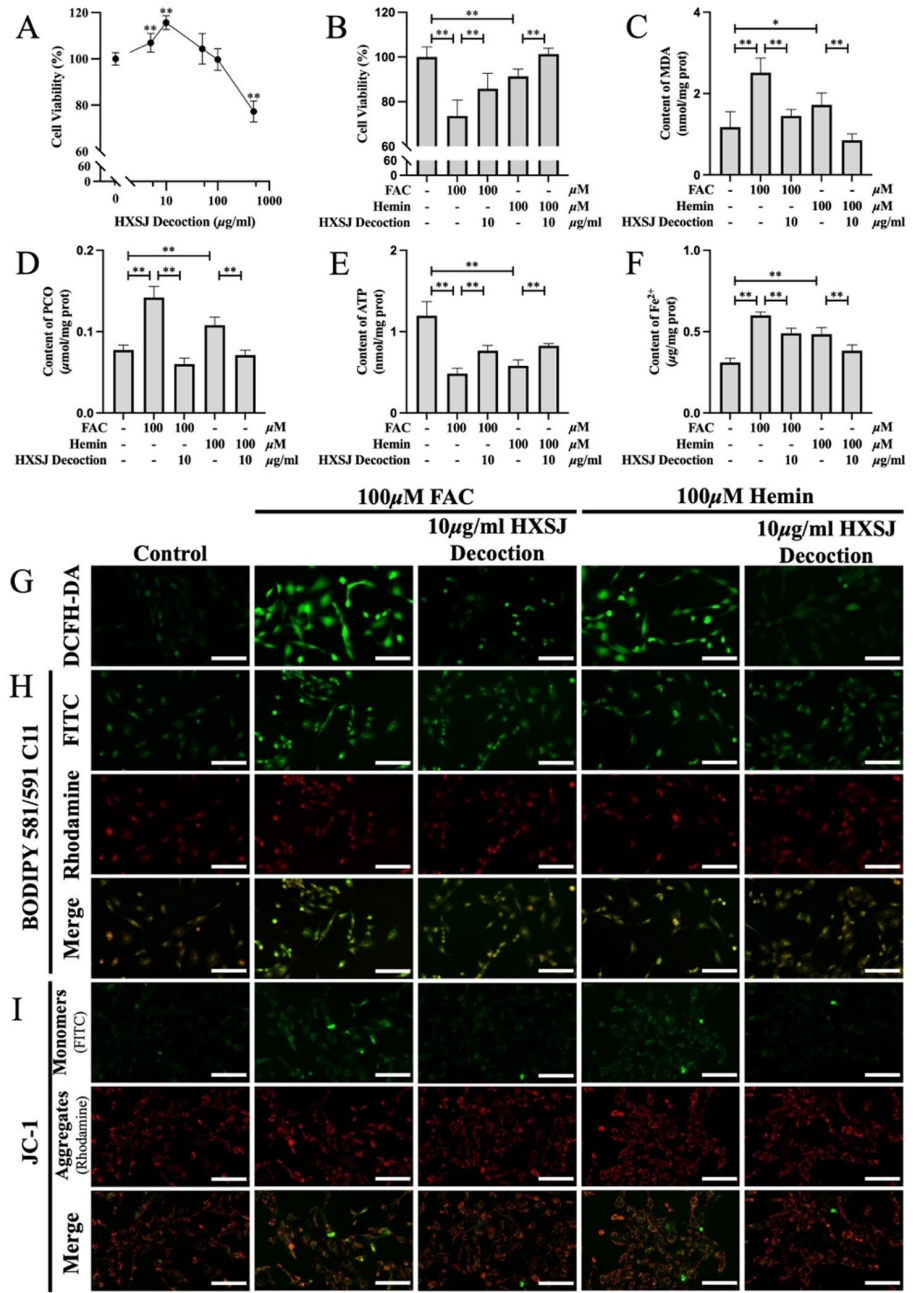
HXSJ decoction mitigated LPO damage and mitochondrial injury induced by iron overload in HUVECs. **A** Effects of HXSJ decoction on HUVEC viability. Cell viability (**B**), MDA content (**C**), PCO content (**D**), ATP content (**E**), and Fe^2+^ content (**F**) in iron overload HUVECs. Fluorescence images of DCFH-DA staining (**G**), BODIPY 581/591 C11 staining (**H**), and JC-1 staining (**I**) in HUVECs; scale bar: 100 μm. **: *P* < 0.01; *: *P* < 0.05; n.s.: not significant

In this study, we investigated the effects of treatment with different concentrations of HXSJ decoction (0–500 µg/ml) for 24 h on HUVEC proliferation. The results of the CCK-8 assay (Fig. 3A) demonstrated that HUVEC viability was most significantly increased at a concentration of 10 µg/ml (*P* < 0.01). However, with further increases in concentration, cell viability gradually decreased in a concentration-dependent manner. Therefore, a concentration of 10 µg/ml was selected for further experiments in this study.

To establish iron overload models, we treated HUVECs with 100 µM FAC or 100 µM hemin. As shown in Fig. 3B, HXSJ decoction significantly restored the cell viability inhibited by FAC or hemin (*P* < 0.01). Additionally, the levels of MDA and PCO, which are characteristic products of LPO damage, were measured. The results (Fig. 3C, D) revealed significant increases in the MDA and PCO levels following treatment with FAC or hemin (*P* < 0.05), indicating that both FAC and hemin induced LPO damage. However, HXSJ decoction significantly reduced the MDA and PCO levels (*P* < 0.01), thereby mitigating LPO damage to HUVECs. These findings were further supported by the results of DCFH-DA and BODIPY™ 581/591 C11 staining (Fig. 3G, H). In addition, both FAC and hemin significantly suppressed ATP generation in HUVECs (*P* < 0.01), and this effect was effectively reversed by HXSJ decoction (*P* < 0.01) (Fig. 3E). JC-1 staining was used to measure the MMP (Fig. 3I), with images showing the repression of the MMP in HUVECs treated with FAC or hemin. In contrast, HXSJ decoction had restorative effects on the MMP. In the iron overload model, excessive Fe²⁺ accumulation was crucial for LPO damage. We quantified the Fe²⁺ content in HUVECs (Fig. 3F) and detected significant increases following treatment with FAC or hemin (*P* < 0.01). HXSJ decoction partially mitigated the accumulation of ferrous ions (*P* < 0.01). Collectively, these results indicate that HXSJ decoction effectively alleviates iron overload-induced damage to HUVECs caused by FAC and hemin.

### Huoxue Shengji Decoction activates the Nrf2/xCT/GPX4 pathway in iron-overloaded HUVECs

**Fig. 4:**
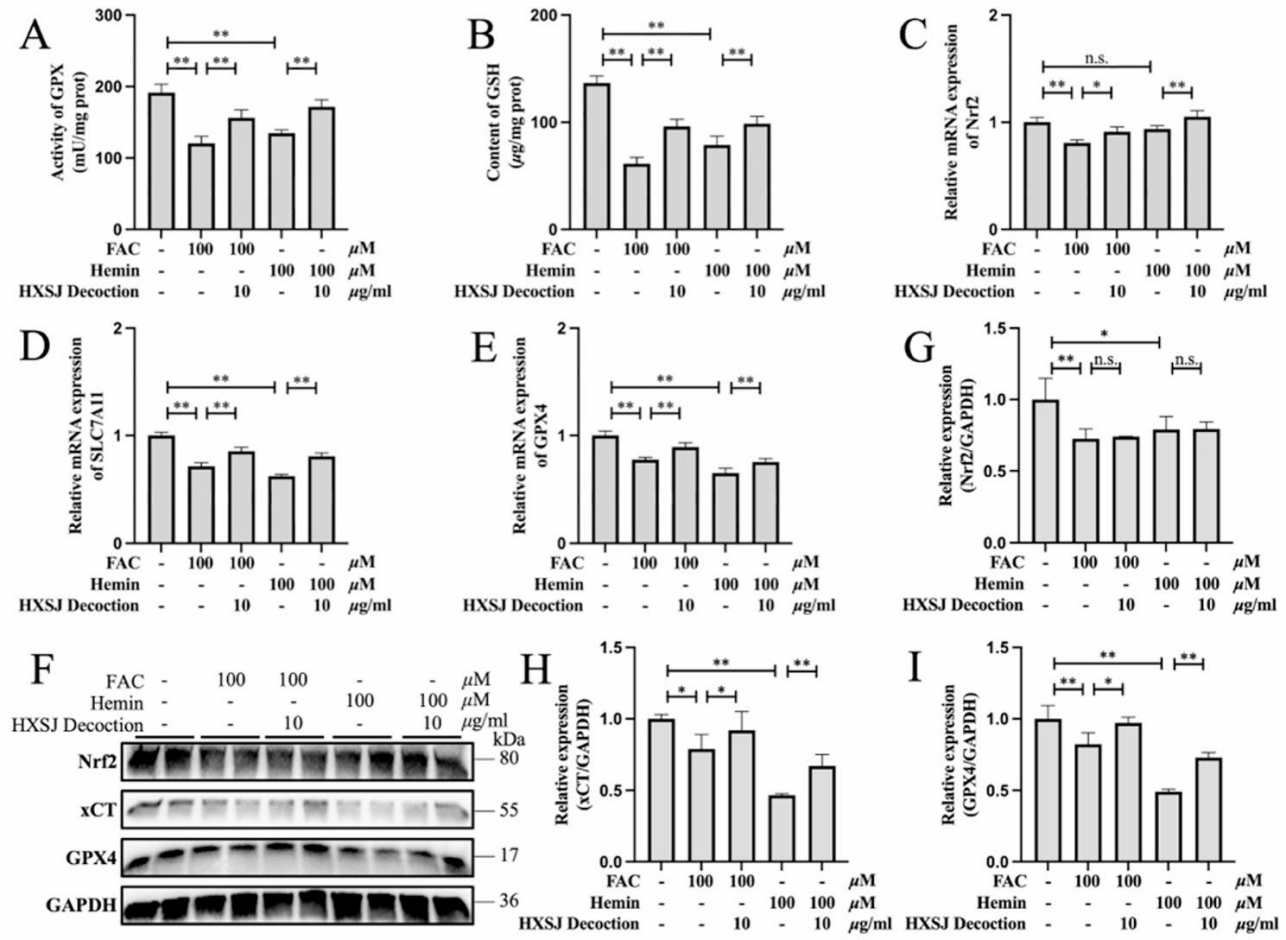
HXSJ decoction activates the Nrf2/xCT/GPX4 pathway in iron overload HUVECs. GPX activity (**A**) and GSH content (**B**) in iron-overloaded HUVECs. Relative mRNA expression of Nrf2 (**C**), SLC7A11 (**D**), and GPX4 (**E**) in iron overload HUVECs. (**F-I**) Western blots of Nrf2, xCT, and GPX4 in iron-overloaded HUVECs. **: *P* < 0.01; *: *P* < 0.05; n.s.: not significant

The Nrf2/xCT/GPX4 pathway plays a pivotal role in defending against LPO damage and ferroptosis. Consistent with these findings, both GPX activity (Fig. 4A) and GPX4 expression (Fig. 4E, F, I) were significantly inhibited in iron-overloaded HUVECs induced by FAC or hemin (*P* < 0.01). As expected, iron overload suppressed Nrf2 (Fig. 4C, F, G) and xCT (Fig. 4D, F, H) expression and depleted GSH in HUVECs (Fig. 4B), although Nrf2 mRNA expression was not significantly downregulated (*P* > 0.05) in the hemin group. Following HXSJ decoction intervention, GPX4 and xCT expression levels, as well as the content of GSH, significantly increased. Although HXSJ decoction promoted Nrf2 mRNA expression, no significant effect on its protein expression was observed. These findings suggest that the mechanism by which HXSJ decoction alleviates ferroptosis in FAC- and hemin-induced HUVECs is associated with Nrf2/xCT/GPX4 pathway activation.

### Huoxue Shengji Decoction mitigates erastin-induced ferroptosis in HUVECs

**Fig. 5:**
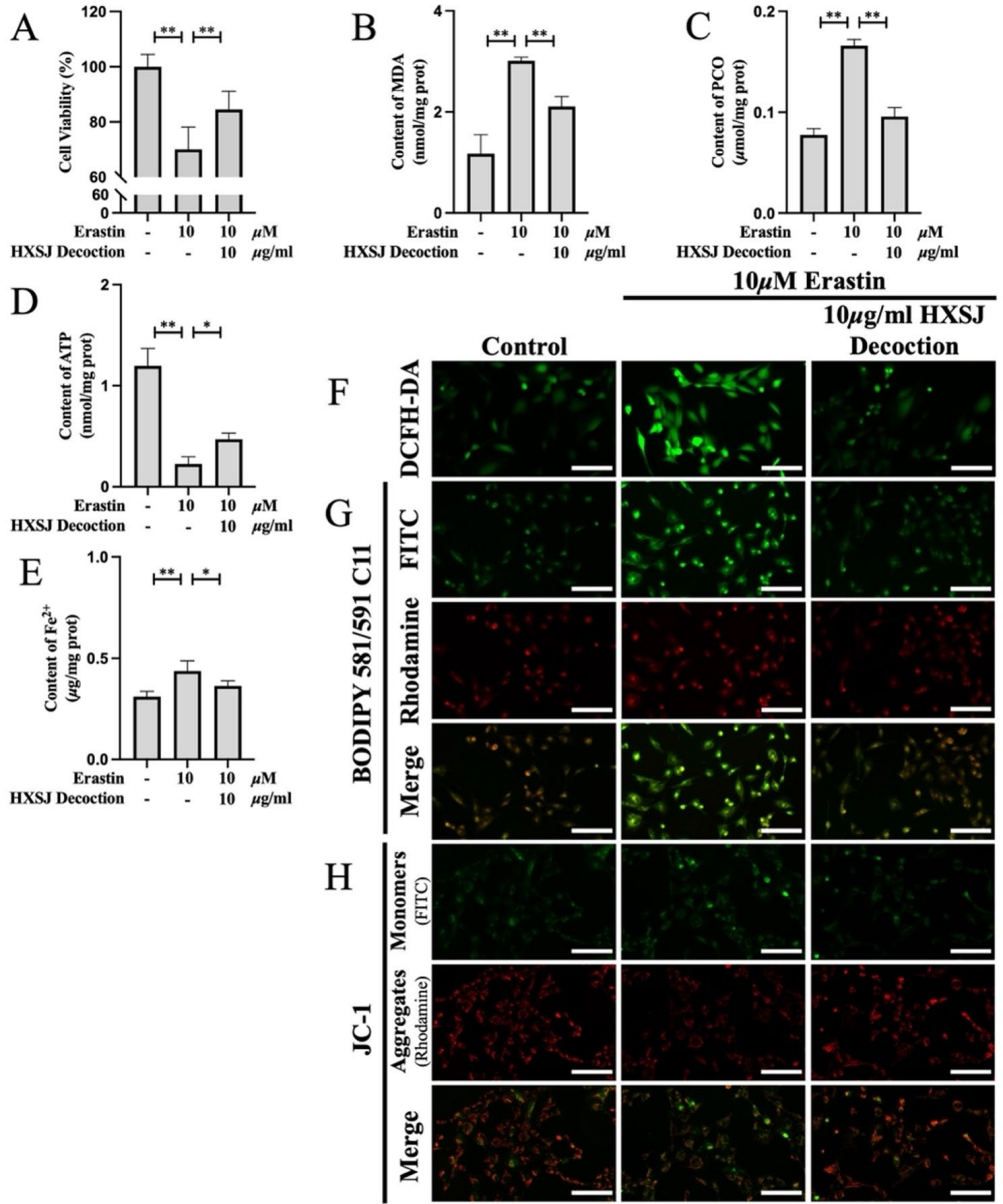
HXSJ decoction alleviates ferroptosis induced by erastin in HUVECs. Cell viability (**A**), MDA content (**B**), PCO content (**C**), ATP content (**D**), and Fe^2+^ content (**E**) in Erastin-induced HUVECs. Fluorescence images of DCFH-DA staining (**F**), BODIPY 581/591 C11 staining (**G**), and JC-1 staining (**H**) in HUVECs; scale bar: 100 μm. **: *P* < 0.01; *: *P* < 0.05; n.s.: not significant

To further investigate the effects of HXSJ decoction on ferroptosis, 10 µM erastin was used to induce ferroptosis in HUVECs. As shown in Fig. 5A, HXSJ decoction significantly restored the cell viability of HUVECs inhibited by erastin (*P* < 0.01). To confirm that the alleviation of erastin-induced ferroptosis by HXSJ decoction occurred, the levels of MDA and PCO were quantified (Fig. 5B, C). HXSJ decoction significantly reduced the MDA and PCO levels in HUVECs treated with erastin (*P* < 0.01). These findings were further validated by DCFH-DA and BODIPY™ 581/591 C11 staining (Fig. 5F, G). Furthermore, HXSJ decoction effectively restored the impairment of mitochondrial function induced by erastin. The depletion of ATP (Fig. 5D) and suppression of the MMP (Fig. 5H) induced by erastin were significantly improved by HXSJ decoction (*P* < 0.01). Although erastin is a commonly recognized system Xc⁻ inhibitor that induces ferroptosis, our study revealed that it also significantly increased Fe²⁺ accumulation in HUVECs (*P* < 0.01) (Fig. 5E). HXSJ decoction partially reduced Fe²⁺ accumulation (*P* < 0.05). Collectively, these results indicate that HXSJ decoction has the potential to mitigate ferroptosis.

### Huoxue Shengji Decoction activates the Nrf2/xCT/GPX4 pathway in ferroptosis in HUVECs

**Fig. 6:**
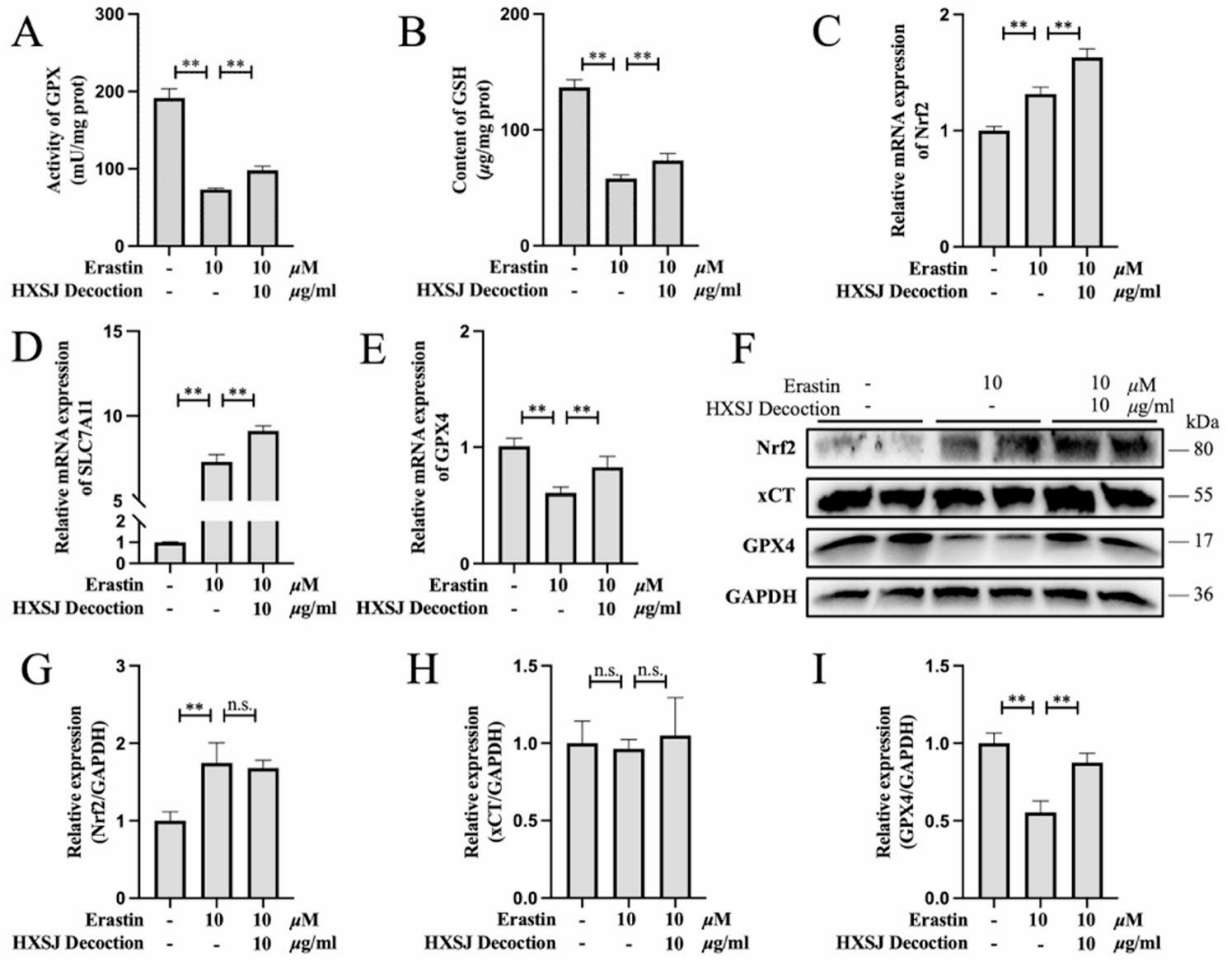
HXSJ decoction activates the Nrf2/xCT/GPX4 pathway in ferroptosis in HUVECs. GPX activity (**A**) and GSH content (**B**) in Erastin-induced HUVECs. Relative mRNA expression of Nrf2 (**C**), SLC7A11 (**D**), and GPX4 (**E**) in erastin-induced HUVECs. (**F-I**) Western blots of Nrf2, xCT, and GPX4 in erastin-induced HUVECs. **: *P* < 0.01; *: *P* < 0.05; n.s.: not significant

As shown in Fig. 6A and B, HXSJ decoction significantly restored GPX activity and increased GSH levels (*P* < 0.01). Further analysis of the Nrf2/xCT/GPX4 pathway (Fig. 6C–F) revealed that erastin significantly downregulated GPX4 expression (*P* < 0.01) and concurrently upregulated Nrf2 expression (*P* < 0.01). Although erastin is recognized as an inhibitor of system Xc⁻, its effect was not evident at the protein level but manifested as significant upregulation of SLC7A11 mRNA (*P* < 0.01). As previously mentioned, HXSJ decoction mitigated LPO damage induced by iron overload through Nrf2/xCT/GPX4 pathway activation, which was also observed in the context of erastin-induced ferroptosis in HUVECs. More importantly, HXSJ decoction upregulated GPX4 expression while further increasing xCT and Nrf2 mRNA levels. These findings suggest that HXSJ decoction exerts its therapeutic effects by alleviating ferroptosis through Nrf2/xCT/GPX4 pathway activation.

## Discussion

Ferroptosis, initially proposed by Dixon in 2012, is a form of programmed cell death that relies on iron overload and LPO [17]. In brief, excessive or unstable ferrous ions contribute to the formation of labile iron pools, generating LPO through the Fenton reaction and inducing ferroptosis [23]. However, even before the concept of ferroptosis was proposed, some researchers had already observed iron deposits in the HPT and LDS regions [7, 8]. In this study, we conducted further research and verified the progressive accumulation of iron during the deterioration of CVD. Interestingly, as the condition progresses to the VLU stage, the iron content significantly decreases. We hypothesize that this may be attributed to the discharge of iron through ulcer exudate, as some studies have reported elevated ferritin levels in chronic ulcer exudate [24]. On the other hand, GPX4 is a key factor through which cells resist ferroptosis, catalysing the conversion of GSH to GSSG and effectively eliminating LPO [25]. Therefore, GPX4 inactivation contributes to ferroptosis, whereas the opposite may alleviate ferroptosis. In our further research, we observed that during HPT–LDS–VLU progression, GPX4 expression exhibited a specific pattern: it increased in the HPT stage, then significantly decreased in the LDS stage, and reached its lowest level in the VLU stage. On the basis of these phenomena, we draw the following conclusions. First, in the LDS stage, GPX4 inactivation and excessive iron accumulation confirm the occurrence of ferroptosis during CVD progression. Second, in the HPT stage, elevated GPX4 expression alleviates LPO damage induced by iron deposition, maintaining a delicate equilibrium between oxidation and antioxidation and thus maintaining pathology at the HPT stage. As iron deposition progressively intensifies or factors such as trauma and infection arise, this delicate equilibrium is disrupted. GPX4 no longer effectively alleviated ferroptosis, leading to the transition to the LDS stage and the inevitable progression to VLU. This speculation elucidates why some patients stabilize indefinitely at the HPT stage, while LDS typically occurs in severe HPT areas; once the condition progresses to the LDS stage, ulcers become almost inevitable. This progression process is intuitively illustrated in Fig. 1. On the basis of these findings, we believe that ferroptosis induced by iron deposition is a trigger for the progression from HPT to VLUs and a persistent barrier to ulcer healing.

Vascular damage plays a pivotal role in ulcer progression and chronicity [5, 6]. In the pathogenesis of VLU formation, sustained venous hypertension induces capillary damage, leading to erythrocyte extravasation. These extravasated erythrocytes are subsequently degraded, releasing haemoglobin and generating haemosiderin deposits, thereby resulting in hyperpigmentation and iron deposition. Iron deposition-induced ferroptosis exacerbates vascular injury, creating a vicious cycle that ultimately drives ulcer formation. In this study, we established iron-overloaded HUVEC models using FAC and hemin (a haem analogue). FAC directly supplies ferric ions, whereas hemin simulates the pathophysiological microenvironment caused by haemoglobin release following erythrocyte degradation in vivo.

The results revealed that HUVECs exhibited ferroptosis, characterized by the inactivation of GPX4, the depletion of GSH, and the accumulation of ferrous ions. Consequently, alleviating ferroptosis in VECs represents a potentially promising therapeutic strategy to improve VLU. Currently, targeting ferroptosis has become a therapeutic strategy in traditional Chinese medicine for disease treatment. We conducted an initial investigation to explore the potential effects of HXSJ decoction on alleviating ferroptosis. HXSJ decoction is an external traditional Chinese medicine compound used to treat VLUs and has been clinically applied by our research group for more than a decade. Our previous clinical research confirmed that HXSJ decoction effectively improved microvascular injury and accelerated ulcer healing [20]. The active constituents of *Astragali Radix*, *Salviae Miltiorrhizae Radix et Rhizoma*, *Carthami Flos*, and *Glycyrrhizae Radix et Rhizoma*, which comprise the HXSJ decoction, include astragaloside [26, 27], salvianolic acid [28], tanshinone [29], carthamin yellow [30] and liquiritin [31], and these compounds exhibit anti-ferroptosis effects. Notably, Nrf2 activation appears to be a common mechanism through which these active constituents exert their anti-ferroptotic effects. As a critical transcription factor in oxidative stress responses, Nrf2 regulates the expression of various target genes essential for alleviating ferroptosis through the modulation of GSH levels, iron metabolism, lipid homeostasis, and mitochondrial function [32]. xCT, also known as SLC7A11, is the active subunit of the cystine/glutamate exchange system (system Xc⁻), providing cystine for GSH synthesis and participating in the process of cellular anti-ferroptosis with GPX4. Therefore, we investigated the effects of HXSJ decoction on alleviating ferroptosis in HUVECs. In this study, 10 µg/mL HXSJ decoction effectively mitigated LPO damage, restored mitochondrial function, and suppressed ferroptosis induced by FAC and hemin via xCT/GPX4 pathway activation. To further validate the activation of the xCT/GPX4 pathway by HXSJ decoction, the ferroptosis inducer erastin was used. Unlike iron overload, which promotes ferroptosis by increasing LPO, erastin primarily induces ferroptosis through two mechanisms: (1) blocking cystine uptake via system Xc⁻, leading to GSH depletion, and (2) inactivating GPX4. Our results demonstrated that erastin downregulated GPX4 expression, accelerated GSH depletion, and upregulated Nrf2 expression. These findings are consistent with those of Lei He et al. [33] and Bei Wang et al. [34]. This seemingly contradictory increase in Nrf2 expression in our study may stem from a negative feedback mechanism that compensates for the suppression of GPX4 expression. Notably, although erastin is widely recognized as a system Xc⁻ inhibitor, we observed no significant inhibition of xCT at the protein level. Instead, erastin markedly increased SLC7A11 (encoding xCT) mRNA expression, which we attributed to Nrf2-mediated transcriptional promotion of SLC7A11 following its activation. Upon treatment with 10 µg/mL HXSJ decoction, ferroptosis in HUVECs was alleviated, and GPX4 expression was restored. Additionally, although HXSJ further elevated Nrf2 and SLC7A11 mRNA levels, it did not significantly affect SLC7A11 protein translation. We hypothesize that this discrepancy arises from two factors: (1) Nrf2 is already in an overactivated state under the effect of erastin, and (2) Nrf2 activity remains regulated by its inhibitor Keap1, and the further increase in mRNA expression may not be reflected at the protein level. On the basis of these findings, we propose that HXSJ decoction alleviates ferroptosis and protects VECs by activating the Nrf2/xCT/GPX4 pathway, thereby repairing vascular injury. This mechanism may significantly contribute to the acceleration of VLU healing.

## Strengths and limitations

Multiple VLUs patients were included in this study. For each patient, skin tissues from the normal, HPT, LDS, and VLU regions were investigated to decrease individual differences among patients. Additionally, it allows the comparison of ferroptosis involvement across different stages from CVD to VLUs. However, the limited availability of tissue samples necessitates caution in interpretation, and the current findings should be regarded as preliminary. In terms of cell experiments, because VLU healing involves complex multicellular interactions, our investigation focused primarily on VECs. Another limitation of this study is that animal experiments were not performed for further validation.

## Conclusions

This study revealed that iron overload and subsequent GPX4 inactivation, which induce ferroptosis, may be critical drivers of VLU pathogenesis. Therefore, ferroptosis inhibition is a promising novel therapeutic strategy for VLU management, with early intervention potentially mitigating disease development. Our data suggest that HXSJ decoction, as a clinically proven traditional Chinese medicine, may facilitate VLU healing by suppressing ferroptosis in VECs via activation of the Nrf2/system Xc-/GPX4 axis. These results support the potential of TCM, particularly HXSJ decoction, as a complementary therapy of VLU.

## Supplementary Information

Supplementary Material 1.

## Data Availability

The datasets used and/or analysed during the current study are available from the corresponding author on reasonable request.

## Abbreviations

VLU: Venous leg ulcer
CVD: Chronic venous diseases
GPX: Glutathione peroxidase
HUVECs: Human umbilical vein endothelial cells
FAC: Ferric ammonium citrate
HXSJ Decoction: Huoxue Shengji Decoction
DCFH-DA: 2’,7’-Dichlorofluorescein diacetate
ATP: Adenosine triphosphate
MMP: Mitochondrial membrane potential
Mrf2: Nuclear factor erythroid 2-related factor
system Xc^−^: cystine/glutamate exchange system
GSH: Glutathione
qPCR: quantitative polymerase chain reaction
VECs: Vascular endothelial cells
HPT: Hyperpigmentation
LDS: Lipodermatosclerosis
LPO: Lipid peroxidation
MDA: malondialdehyde
PCO: protein carbonylation
ROS: reactive oxygen species
SLC7A11: solute carrier family 7 member 11
