# Supplementary material for: Potential mechanism underlying HXSJ decoction in the treatment of venous leg ulcers: based on the association between venous leg ulcers and ferroptosis
Source: BMC Complement Med Ther. 2025 Dec 30;25:451. doi: 10.1186/s12906-025-05184-3 (PMC12751643; doi:10.1186/s12906-025-05184-3)
Supplement: Supplementary file 1 — Supplementary Material 1. [file 12906_2025_5184_MOESM1_ESM.pdf]

Fig4

The left image is the WB marker used in this study. The middle image is he result of horizontally excising the same membrane, followed by antibody incubation (merging bright-field and fluorescence images). The right image is the original WB fluorescence image.

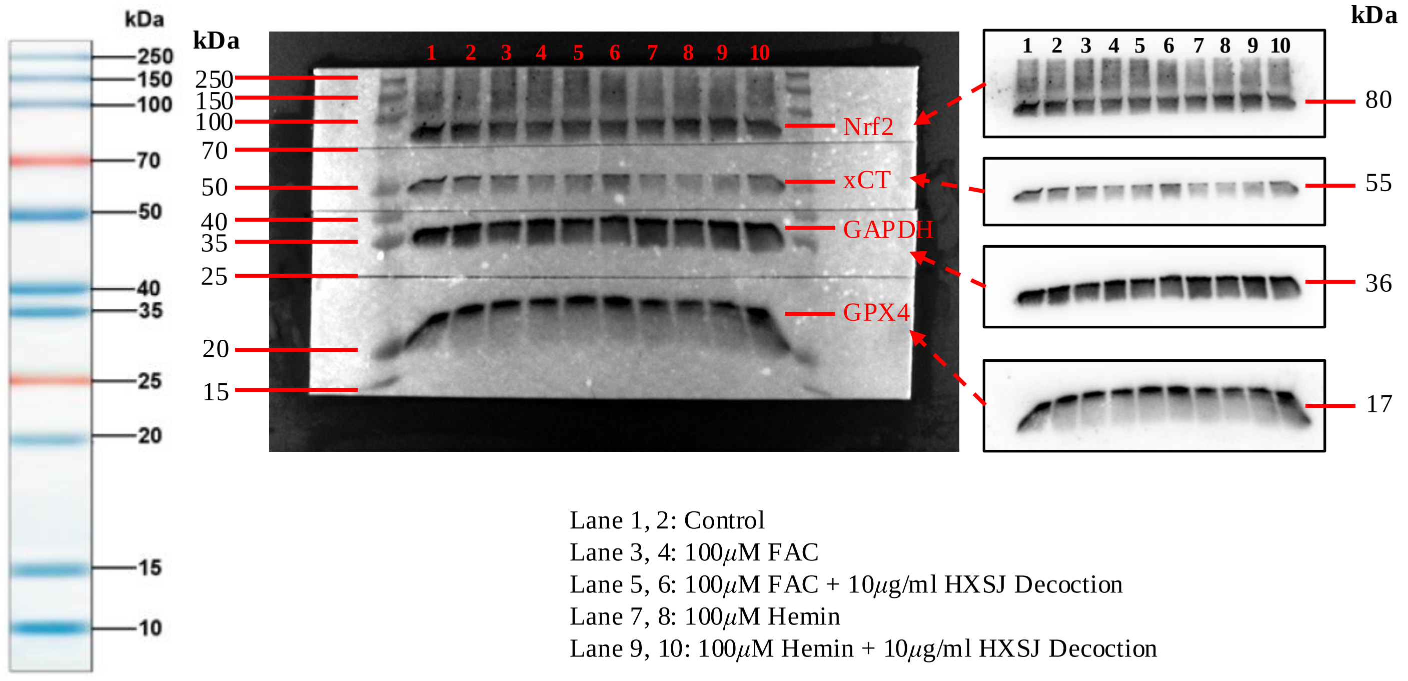

Lane 1, 2: Control
Lane 3, 4: 100μM FAC
Lane 5, 6: 100μM FAC + 10μg/ml HXSJ Decoction
Lane 7, 8: 100μM Hemin
Lane 9, 10: 100μM Hemin + 10μg/ml HXSJ Decoction

Fig6

The left image is the WB marker used in this study. The middle image is he result of horizontally excising the same membrane, followed by antibody incubation (merging bright-field and fluorescence images). The right image is the original WB fluorescence image.

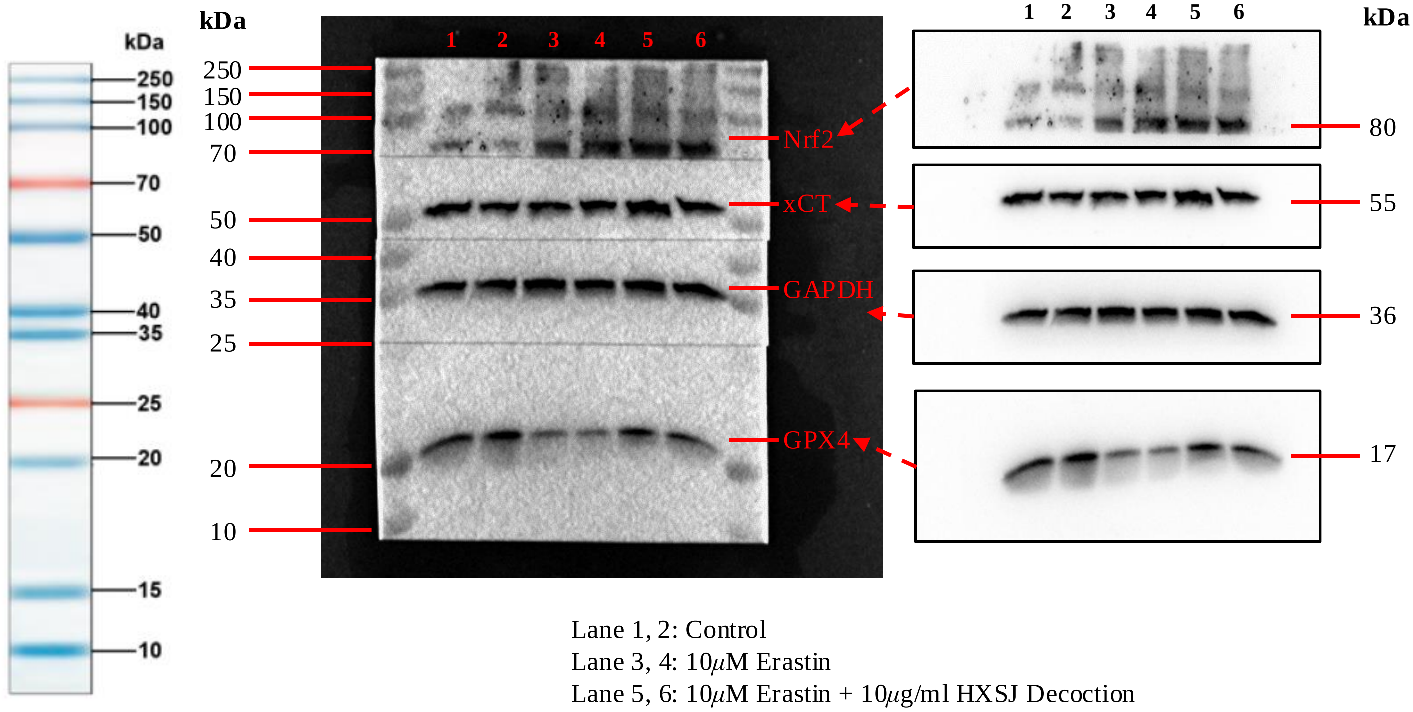

Lane 1, 2: Control
Lane 3, 4: 10μM Erastin
Lane 5, 6: 10μM Erastin + 10μg/ml HXSJ Decoction
